# The impact of Otago exercise programme on the prevention of falls in older adult: A systematic review

**DOI:** 10.3389/fpubh.2022.953593

**Published:** 2022-10-20

**Authors:** Yi Yang, Kun Wang, Hengxu Liu, Jiawei Qu, Yan Wang, Peijie Chen, TingRan Zhang, Jiong Luo

**Affiliations:** ^1^Research Centre for Exercise Detoxification, College of Physical Education, Southwest University, Chongqing, China; ^2^School of Physical Education, Sichuan Agricultural University, Yaan, China; ^3^Leisure College of Shanghai Institute of Physical Education, Shanghai, China

**Keywords:** Otago exercise programme, prevent falls, balance ability, cognitive function, fall efficiency

## Abstract

**Objective:**

To improve the quality of life of older adult in their later years, by increasing the physical activity participation of older adult, the occurrence of falls accident scores in older adult can be prevented. This paper comprehensively summarizes the origin, development, participation forms, and fitness effects of the Otago exercise program (OEP).

**Methods:**

Using PubMed, web of science, CNKI, dimensional spectrum, and other databases, search for research papers from 2005 to April 2021 by using keywords such as Otago project exercise; aged, Fall; Cognitive function, Balance ability, Lower limb strength, Fall efficiency, and so on. PEDro Scale was used to check the quality of the literatures.

**Results:**

A total of 34 papers were included after searching for kinds of literature related to the subject of this paper and after careful review by researchers.

**Conclusions:**

Otago exercise programme is beneficial to improve the cognitive function of older adult, enhance their lower limb muscle strength and dynamic and static balance ability, and then improve the gait stability and posture control ability of older adult, which has significant positive benefits for the prevention of falls in older adult. OEP is helpful to improve the falling efficiency of older adult, help older adult overcome the fear of falling, and form a positive emotion of “exercise improves exercise,” to reduce the harm caused by sedentary behavior and the incidence of depression and improve their subjective wellbeing. Although OEP has significant positive effects on improving the health and physical fitness of older adult, preventing falls, and restoring clinical function, the corresponding neural mechanism for preventing falls is not very clear. At the same time, how OEP can be combined with emerging technologies to maximize its benefits needs to be further discussed in the future.

## Introduction

Falling refers to falling on the ground or below the level without conscious or external force (1). It is easy to cause fracture, stroke, and limited mobility in older adult. Indirectly, it causes physical weakness, cognitive decline, sedentary behavior, social exclusion, and even death (2, 3). Aging leads to the decline of balance ability and posture control ability of older adult, which increases the risk of falls, About 300,000 people worldwide die from falls every year. Among older adult over 65 years old, 30% have fallen and 15% have fallen many times (4). Therefore, the injury caused by falls has become an important public health problem, which has a huge negative impact on the high-quality and healthy life of older adult.

As we all know, human body function and neurosensory perception will weaken with age, accompanied by chronic diseases or sarcopenia and other diseases; At the same time, aging causes problems such as long reaction time, cognitive decline, balance ability, muscle strength, and side effects of drugs, which will increase the risk of falls (5, 6). However, proper participation of older adult in sports events focusing on flexibility, lower limb strength, and balance can improve the balance ability and physical flexibility of older adult and reduce the occurrence of fall injury (7, 8). According to relevant research reports, at present, many exercise prescriptions are effective in preventing falls in the world, such as the Otago exercise program (OEP), fitness exercise for older adult, fall prevention lifestyle, multi-objective stepping exercise, Tai Chi, yoga, Pilates and resistance training (9). Among them, OEP has been proved to effectively improve the cognitive function, balance ability, lower limb muscle strength, functional physical fitness of older adult (10), prevent falls in older adult (11), accelerate the recovery of physical function, and reduce economic costs (12, 13), because its training content emphasizes strength and balance exercises more; In recent studies, it was also found that OEP can not only effectively improve the balance ability, but also enhance the self-confidence of balance control after multiple falls and overcome mental health problems such as social isolation and fear (14, 15). In short, with the aggravation of population aging, more and more elderly people cannot go out to participate in leisure activities due to physical reasons, and can only stay at home for a long time due to physical reasons (such as arthritis, stroke, heart disease, and other chronic diseases), poor health self-assessment, and less social support and higher living floors. Staying at home during the period can reduce the amount of physical and mental activity of older adult, reduce their mobility and balance, and increase the risk of falls. Falls may lead to the decline of the quality of life of older adult and the improvement of the bedridden rate. These serious clinical problems will greatly increase the family and socio-economic costs. The Otago exercise program aims to prevent falls in older adult, the personalized and progressive exercise of muscle strength and balance at home is just a multi-component exercise prescription of personalized and progressive exercise of lower limb muscle strength and balance at home. Therefore, this review will comprehensively comb and summarize the content, development history, and fitness efficacy of OEP, to better understand and promote OEP, help older adult actively deal with falls, and provide a theoretical basis for future research design and practical application.

## Date and methods

### Date sources

Using PubMed, web of science, China National Knowledge Infrastructure (CNKI), dimensional spectrum, and other databases, search for research papers published in relevant journals at home and abroad from 2005 to April 2021 by using keywords such as Otago project exercise; aged, Fall; Cognitive function, Balance ability, Lower limb strength, Fall efficiency, Subjective wellbeing, self-confidence and so on.

### Eligibility criteria

(1) The study group consisted of older adults 60 years and older who were at risk of falls or had a history of falls. (2) The experimental group had a strict exercise prescription design. (3) The exercise prescription of the experimental group must be based on the OEP, and the control group can be prescribed other exercises or not intervened. (4)The prescription design is following the standards of the American College of sports medicine (ACSM). The evaluation indexes mainly include cognition, balance ability, lower extremity muscle strength, and fall efficacy.

### Literature exclusion criteria

(1) Literature whose language is not English or Chinese is excluded. (2) Repeated and nonexperimental studies were excluded. (3) An experimental study to exclude Otago exercise prescriptions unrelated to the subject of falls.

### Data intake quality assessment

1. The reading and review of the literature are divided into three stages. In the first stage, The researcher searches the literature in the database, and initially browses and reads the abstract to select the appropriate literature. In the second stage, other researchers sorted out the literature and eliminated duplicate literature. In the third stage, two researchers jointly read the full text to determine whether the articles met the inclusion criteria. If there is any literature that has not reached a consensus, it will be decided after discussion.
2. Literature quality and empirical level. PEDro scale was used to check each document and evaluate its research quality. The higher the score, the better the research quality of this document. Each document was scored independently by two researchers. If there are different scoring items, a consensus was reached after discussion. Due to the characteristics of the included papers, the therapists are required to provide treatment intervention in the research process. The highest total score maybe 9 for the items that cannot be single-blind for the therapists. Therefore, it is determined that those whose pedroscale score is ≥5 are high-quality papers, and those whose score is ≤4 are low-quality papers.

**Figure 1:**
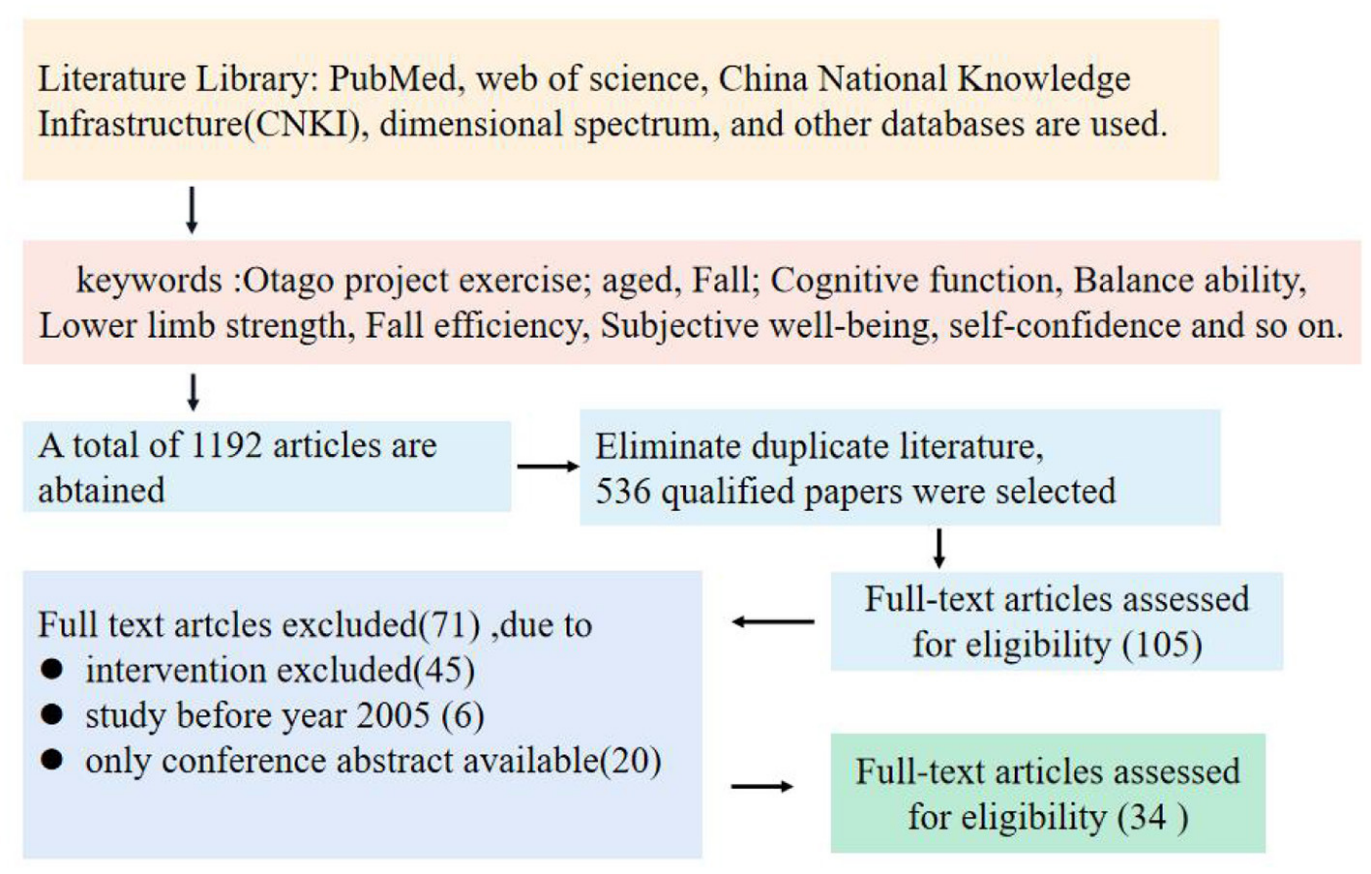
Search and exclusion process flow diagram.

The system search results are shown in Figure 1. A total of 1,192 relevant articles were retrieved from four databases. Deleted 656 duplicate literature, and selected 536 kinds of literature According to the title and abstract, 105 full texts were obtained for further analysis, of which 71 were excluded because they did not meet the qualification criteria. Through the full-text analysis, 34 papers meet the qualification criteria. The number of articles finally included in this paper is 34.

## Results

### Origin and development of Otago exercise program

The OEP originated in New Zealand, Campbell et al. (16) conducted the intervention pilot study of this exercise therapy for elderly women for the first time and achieved good results. OEP has now been listed as a key intervention project for fall prevention in New Zealand and has invested a lot of money in development and promotion (17). The training content of OEP is mainly composed of four parts: warm-up activities, strength training, balance training, and walking training, 5-min warm-up activities: head movement, neck movement, back stretching, trunk movement, and ankle movement; Strength training and balance training take about 30 min, of which five strength training include sitting knee extension, standing hip abduction, standing knee flexion, tiptoe and heel tiptoe; The 12 balance exercises include standing on one foot, walk in the shape of the number eight, walking sideways, walking backward, standing to sit position training, knee bending, toe to heel standing, heel walking, toe to heel walking, toe to heel walking, toe to heel walking, toe to heel walking backward, and climbing stairs; The last part consists of 10-min walking training to consolidate the effect of muscle strength and balance training. The exercise intensity is divided into four levels of ABCD, and the intensity increases gradually; The exercise frequency shall be no <3 times a week. In terms of training monitoring, physiotherapists understand the training status of older adult and adjust the content through telephone interviews and home visits (11, 18, 19).

After the initial success of OEP in New Zealand, it was gradually introduced by Germany, the United States, Canada, and other countries, and OEP was improved and developed according to the actual situation. American scholars have discussed the enforceability and scientificity of OEP and demonstrated it many times in combination with the feedback of physiotherapists. The research results preliminarily prove the effectiveness of OEP, but there are still some problems, such as imperfect rehabilitation training institutions, lack of professional guidance, and high medical expenses (20). Subsequently, the improved OEP gradually became efficient and feasible. Through the training of professional physiotherapists and combined with virtual technology, its feasibility and scientificity were ensured (11, 21). OEP has been widely used in clinical rehabilitation and postoperative recovery. The intervention objects are stroke (22), knee arthritis (23), cognitive impairment (24), and knee replacement surgery (25). After OEP intervention, the patients' physical function and balance ability have been effectively improved, and their fall risk has also been effectively reduced.

The operation form of OEP training can take the form of personal exercise, emerging technology, and group exercise. To carry out the traditional mode of OEP, professional physiotherapists need to be equipped to design movement guidance and exercise prescriptions, and fully grasp the comprehensive situation of training (26). With the development of multimedia technology and intelligent platform, the form and operation of OEP are becoming more and more intelligent and diversified, such as augmented reality (VR), remote live broadcasting, and wearing trackers (12, 27). older adult living alone can complete exercise tasks online. Physiotherapists use wearable devices to monitor the physical condition and training of exercisers, and give established plans according to the actual situation, At the same time, online OEP training can reduce the sense of loss of older adult due to differences in skill levels and enhance their confidence in adhering to sports (28), To maximize the satisfaction of the personalized needs and sense of security of older adult at home, it is very effective to use such a way of exercise that breaks through the limitations of time and space in the period of epidemic isolation. However, some studies believe that OEP in group mode expands the social participation of older adult and has stronger compliance than exercise alone (7). In short, OEP is beneficial to older adult. No matter what form of exercise OEP is, it is effective in specific circumstances.

### Effect of OEP on the prevention of falls in older adult

#### Effect of OEP on cognitive function of older adult

The most common mental disorders of older adult mainly include dementia, depression, delirium, etc., commonly known as 3D diseases, These three factors often lead to the functional decline of older adult in a short time, which not only leads to the impairment of older adult's self-care ability, but also gives caregivers a great burden and increases the difficulties in care, and the resulting complications increase the medical cost.

Otago exercise program training can improve the brain processing speed, sustained attention, visuospatial skills, working memory, and other related cognitive abilities of older adult (24). In terms of executive function, as a key field of cognitive function, the advanced cognitive process of controlling and integrating other cognitive abilities can test and evaluate the fall risk level of older adult (29). The role of executive function in preventing falls can be achieved through exercise induction (30). After exercise therapy, the improvement of executive function will help to improve the compliance of older adult and better adhere to participate in the exercise. It is worth noting that OEP has positive effects on motor function in improving neurocognitive function in older adult, and there is also an interaction between neurocognitive function and motor function (31). According to the special physiological conditions of patients with mild cognitive impairment, the weak state further increases their fall risk. The group mode OEP is a potentially effective strategy to improve patients with mild cognitive impairment, which can improve their cognitive weakness to a certain extent (32, 33). In addition, after OEP intervention, it was found that cognitive status and activities of daily living at baseline can predict the longitudinal pattern of compliance, and a higher baseline level of executive function may lead to better use of self-regulation strategies, which is beneficial to the prevention of falls in older adult (34). Maintaining and improving executive function plays a long-term role in exercise. The improvement of working memory and attention can also promote older adult to adhere to exercise and reflect good compliance (35). However, these three studies only prove that OEP can improve the physical function of older adult and indirectly reflect the positive effect of cognitive function on older adult. There are some limitations because it is not studied directly from the cognitive level. It may be more valuable to explore the research on reducing the risk of falls directly from the cognitive level of exercise improvement. Response inhibition is an important part of executive function, which mainly includes intentional inhibition of dominant response, automatic response, or strong response. After 6 months of OEP intervention, the lower limb strength and balance ability of older adult were improved, and the most important thing was to improve the path of response inhibition, which proved the effectiveness of preventing falls and reducing the risk of falls in older adult by improving cognitive performance (36). OEP is widely used in rehabilitation training for special patients. Stroke patients will be at risk of cognitive impairment due to factors such as cerebral ischemia and brain damage. Studies have shown that OEP combined with music therapy can improve the memory ability, orientation ability, and language ability of stroke patients, contribute to the repair of neurological function and improve cognitive impairment (37). In addition, short-term Ortega exercise can not only improve the cognitive function of stroke patients, but also greatly improve their ability of daily living, and then promote the improvement of the quality of life of elderly stroke patients (38). The research data for this part are shown in Table 1.

**Table 1:** Research data on OEP improving cognitive function in older adult.

**Researcher**	**Country**	**Sample size**	**Intervention time**	**Intervention mode**	**Test task**	**Research results**
Liu-Ambrose et al. (24)	Canada	*n* = 256; Age ≥ 70	1 year, 3 times a week/30 min	①OEP ②Routine nursing	MOCA; MMSE	The score of the digit symbol replacement test in the intervention group was higher than that in the control group, indicating that the processing speed of older adult was effectively improved after the intervention
Huang and Ou (31)	China	*n* = 87; Age ≥ 60	3 months, 2–3 times a week/30 min	①OEP ②Routine nursing	NIS	After OEP intervention, neurological function was improved based on improving motor function
Davis et al. (33)	Canada	*n* = 172; Age ≥ 70	1 year, 3 times a week/30 min	①OEP ②No	MOCA; MMSE	The average score of recommended mental state after the intervention was 27.7 (24–30 were normal), and the improvement of cognitive function reflected good compliance
Liu-Ambrose et al. (35)	Canada	*n* = 74; Age ≥ 70	6 months, 3 times a week/30 min	①OEP ②Routine nursing	MMSE	There was a significant difference in response inhibition between the OEP group and control group (*p* < 0.05); The risk of falls was 0.47 in the OEP group and 0.56 in the control group
Liu et al. (36)	China	*n* = 142; Age ≥ 60	6 months, 3 times a week	①OEP ②Routine nursing	MMSE	After OEP intervention, the memory ability and orientation ability of stroke patients were significantly higher than those before intervention and those in the control group
Davis et al. (34)	Canada	*n* = 172; Age ≥ 70	1 year, 3 times a week/30 min	①OEP ②Routine nursing	ODT; MOCA	The improvement of executive function, attention, and working memory promote older adult to adhere to exercise and reflect better compliance
Shao (37)	China	*n* = 30; Age ≥ 70	6 months, 3 times a week/30 min	①OEP ②Routine nursing	MOCA; MMSE	After OEP intervention, the left and right hippocampal connections changed, and the scores of delayed memory, attention, and language were significantly improved (*p* < 0.01)

① experimental group ② control group.

The intervention time is only the intervention time of the experiment.

M0CA, Montreal Cognitive Assessment; MMSE, simple mental state score; NIS, National Institutes of Health Stroke Scale; ODT, Oral digital test.

#### Effect of OEP on balance ability of older adult

##### Effect of OEP on static balance ability of older adult

Static balance ability can keep older adult stable in a special environment, such as sitting and standing posture. It seems that static posture is not easy to fall. But, on the contrary, when older adult are in a sitting or standing position, poor physical control will lead to falls and backward tipping, resulting in physical injury. In a previous static balance study of 68 elderly people, it was observed that after 12 weeks of OEP intervention (39), the Berg Balance Scale (BBS) scoring ability was significantly improved, and the balance scale score increased from 15.32 ± 2.18 to 16.78 ± 2.20, and the static balance ability of older adult was significantly improved (*p* < 0.001). The risk of falls decreased from two times before intervention to 0 times. In addition, the physical fitness level of older adult in older adult nursing home has also been greatly improved (1). Current research reports that online OEP intervention in sedentary elderly can effectively improve their static balance ability, and its economic cost is not high. The most important equipment cost is wearable tracker equipment, which is used to measure basic information, and the content of OEP is presented in the form of a video conference (27). After the balance training and walking exercise program based on family basic OEP, the number of falls and disability risk of older adult in assisted living facilities are reduced (40, 41).

The OEP of virtual mode seems to be more in line with the realistic requirements of the rapid development of science and technology. OEP training based on virtual reality improves static posture control and can achieve the training effect of traditional OEP. This method is beneficial to older adult with special contraindications, such as home-based elderly with mobility difficulties, asthenia, etc (42). After standing training through the new virtual OEP, the single leg standing test index and the 30-s sitting test score are higher than the pre-test score, which shows that the static balance ability of older adult has been improved and the fall risk has been improved in the objective measurement index (20), However, there are some limitations. This form of OEP has poor benefits for the weak elderly; Recent empirical studies have proved that group OEP in virtual mode can reverse the frailty of elderly patients with mild cognitive impairment and the physiological function of cognitive impairment, and improve their balance ability (43) and physical activity ability (32). However, different intervention objects will lead to some differences in research results.

##### Effect of OEP on dynamic balance ability of older adult

Dynamic balance ability is an important guarantee for older adult to step out of the small home environment. older adult need better dynamic balance ability to maintain posture stability in their daily activities. Multi-component exercise can improve older adult's posture control ability, maintain certain stability in moving activities such as climbing stairs, and prevent them from falling, causing damage and affecting their quality of life. Relevant studies have reported that short-term group OEP can effectively improve the balance ability of older adult, improve the physical level of older adult, and enable older adult to obtain good compliance and satisfaction (44); After repeated training such as sitting and standing, standing and walking, the dynamic balance ability is significantly improved (39). Shubert te et al. (11) found through the comparative study of the two modes of family OEP and community OEP that both modes improve the balance ability of older adult and have positive benefits for older adult, but there are differences in economic level and development mode. The OEP of group mode can participate more elderly people, and the economic cost is more affordable than private treatment, but the positive effect of the traditional mode on special elderly people is indispensable. Therefore, OEP can be used as a long-term treatment for older adult to prevent falls, meet the diversified needs of older adult and improve their actual balance ability. As a traditional Chinese national sport, Tai Chi has a long history and has the effect of cultivating the body and mind and strengthening the body, and is deeply loved by older adult. Tai Chi and OEP are both effective means to prevent falls in older adult. Some scholars compared the two methods and found that after participating in Tai Chi and OEP intervention, both interventions improved the balance ability of older adult, but the balance ability of the OEP intervention group was better. The test indexes of sitting and standing in 30 s and step frequency were significantly higher than those of the Tai Chi Group, but the test index of standing on one foot of the Tai Chi group was better than that of the OEP group, This may be due to the different exercise modes and muscle use modes of the two. There are more balance training contents in OEP, especially dynamic training, while tai chi moves more slowly with one foot, but both improve the dynamic balance ability of older adult on the whole (8). In the OEP plan, walking backward on tiptoe and walking eight characters have improved the coordination and flexibility of older adult, and further increased the extension length and stability of older adult during movement (45). The combination of the movement observation method and OEP increases the understanding and excitement of movement in relevant brain areas such as the motor cortex, and shows the improvement of balance ability and walking speed in practical performance (46). The research data for this part are shown in Table 2.

**Table 2:** Research data on OEP improving balance ability.

**Researcher**	**Country**	**Sample size**	**Intervention time**	**Intervention mode**	**Test task**	**Research results**
Jahanpeyma et al. (1)	turkey	*n* = 72, Age ≥ 65	12 weeks/3 times/45 min	①OEP ② None	Berg balance scale; 30-s standing test	After the intervention, the score on Berg Balance Scale was significantly improved (*p* < 0.001), and the number of falls was significantly reduced (*p* < 0.005)
Beato et al. (39)	America	*n* = 30 Age ≥ 60	9 weeks/3 times/30 min	①OEP ② None	Manual muscle strength test; Time stand	After OEP intervention, the average number of falls per person per year decreased from 1.4 to 0.5; The physical performance score increased from 11.8 to 17.6
Knott et al. (40)	America	*n* = 59 Age ≥ 70	Unknown	①OEP ② None	Mobile capability test; Balance ability test	After the intervention, the number of falls was significantly improved, and the balance ability and mobility index was increased
Phu et al. (41)	Australia	*n* = 195 Age ≥ 70	6 weeks/twice/60 min	①OEP ② None	30-second standing test; Square step experiment	The posture stability and gait speed of older adult in the OEP intervention group were higher than those in the control group (*p* < 0.05)
Feng et al. (43)	China	*n* = 18, Age ≥ 65	12 weeks/3 times/60 min	①OEP ② None	30-second standing test; Four-stage balance test	After the intervention, the post-test vulnerability score was significantly lower than the pre-test score (*p* < 0.05), and the physical performance and static balance ability were improved
An et al. (44)	China	*n* = 32, Age ≥ 65	21 weeks/3 times/30 min	①OEP ② None	Balance ability test	After the intervention test, the physical fitness and balance level of older adult increased significantly, and the group OEP has good adaptability
Chen et al. (38)	China	*n* = 60, Age ≥ 65	12 weeks/3 times/45 min	①OEP ② None	Berg balance scale; Four-stage balance test	The static and dynamic balance of older adult were improved (*p* < 0.001), and their physical function was enhanced
Shubert et al. (11)	America	*n* = 210 Age ≥ 65	8 weeks/day/3 times/30 min	①OEP ② None	30-second standing test; Four-stage balance test	After the intervention, they significantly improved their physical objective function, flexibility, balance ability, and self-report ability
Son et al. (8)	Korea	*n* = 46 65–79	12 weeks/twice/30 min	①OEP ②Taichi	Functional extension test; One leg standing test	Both groups improved the mobility of older adult, which is conducive to the prevention of female elderly falls
Liew et al. (45)	Malaysia	*n* = 67; Age ≥ 65	12 weeks/3 times/35 min	①OEP ②Routine nursing	Lower limb function test Hand dynamometer	OEP compared with the control group, the increase of upper limb grip strength and balance ability further enhanced the posture control ability of older adult
Leem et al. (46)	Korea	*n* = 30; Age ≥ 70	12 weeks/3 times/50 min	①OEP ② Action observation	Standing walking test	OEP improves balance and walking speed in actual performance

① Experimental group.

② Control group.

#### Effect of OEP on lower limb muscle strength in older adult

Otago exercise program improves the lower limb strength of older adult through its special training content, to reduce the risk of falls older adult. OEP's muscle training is a kind of repeated low-intensity resistance training, which stimulates knee flexion, anterior tibial muscle, and ankle dorsiflexion, fully activates the muscle, maximizes the performance ability of muscle fibers, and may increase the synthesis of actin and myosin, thus delaying the atrophy of muscle cross-sectional area and the decline of muscle strength (12). In a study on grip strength through OEP, it was found that 6 months of lower limb training and exercise can significantly improve the grip strength of older adult, which is in line with the cross effect of exercise and helps to prevent sarcopenia and decline of muscle strength in older adult (45). Although OEP can increase the lower limb muscle strength and reduce the risk of falls and fractures in older adult, some studies believe that this exercise mode cannot increase bone mineral density, which may be due to the time of intervention and the failure of exercise load to reach its threshold (47). A study used an electronic muscle strength meter to measure the effect of OEP on muscle strength and found a positive effect. The “tiptoe” and “tiptoe heel” exercises in OEP stimulate the stretching of lower limb muscles, which may help to increase the body's demand for protein, promote the increase of muscle protein synthesis and muscle oxygen consumption, increase muscle content and improve lower limb muscle strength. Long-term living alone or sedentary behavior can lead to depression and mental health problems, and depression can reduce interleukin-6 and tumor necrosis factor α The levels of pro-inflammatory factors such as C-reactive protein are increased (48), which will lead to the decline of muscle density and skeletal muscle quality. OEP can reduce pro-inflammatory factors to improve the depression of older adult and slow down the decline of muscle function in older adult (49).

Lower limb strength is the basic guarantee of mobility. The lack of lower limb strength will lead to slow movement and a limited range of activities for older adult, which is one of the reasons why many elderly can only stay at home. After OEP intervention, the mobility of older adult can be improved, which can increase their social participation and sports participation. In the form of group OEP, older adult in the nursing center were taken as the research objects. OEP was conducted three times a week for 6 months, with a total of 78 training sessions, After the intervention, the results showed that the strength of the ankle muscle group and ankle dorsiflexion increased significantly. The muscle strength increased from 7.02 to 12.92 kg before the intervention, which increased the strength of the lower limbs. Compared with the pre-test and post-test data for the sitting and standing test, the number of sitting and standing in the experimental group increased from 5.11 ± 2.57 to 9.33 ± 5.12. There were no falls and adverse events during the 6 months of the intervention. The exercise prescription did not need special equipment, Therefore, scholars believe that OEP is a simple, safe, and effective lower limb resistance exercise (50). In the contrast to augmented reality-based OEP and yoga, OEP effectively improved the knee flexion and ankle dorsiflexion strength of elderly women (12). Another study also supports this view that OEP improves the lower limb strength and physical fitness level of older adult, but the control group is the walking exercise group, and the OEP itself includes walking exercise. There is no significant difference between the two, but this does not affect its positive effect (1). Similarly, OEP is efficient and safe to improve the lower limb strength of older adult under special physiological conditions. Compared with Taijiquan, the OEP plan includes many resistance training for lower limbs to overcome self-weight, which is better than Taijiquan in improving specific lower limb strength, such as ankle strength, knee extension, and flexion strength (8). The nursing home is also an effective model for older adult to improve their muscle weakness and long-term muscle function (45).

Otago exercise program effectively improved the lower limb muscle strength of patients with a history of falls and stroke. Some scholars have discussed the positive benefits of OEP in older adult with knee arthritis disease. The results show that OEP still has a positive effect on the lower limb muscle strength of older adult with knee arthritis, and there is no adverse effect on knee arthritis (23). Although the research results are not fully sure to effectively reduce the fall recurrence rate of older adult with a special history, it can benefit older adult with gait disorders; older adult often suffer from chronic diseases, including lower limb muscle pain, which will hinder the exertion of lower limb muscle function. After OEP, the pain was relieved. It is speculated that lower limb training may improve muscle performance (26), but some studies have found that although OEP can significantly improve the lower limb muscle strength of older adult with another special medical history, it has little effect on their self-care ability, which may be related to the special medical history. In the study on fall prevention of stroke patients, an OEP plan can significantly improve the lower limb strength of stroke patients and reduce the risk of falls, with little impact on their activities of daily living and quality of life (51). OEP can increase the lower limb strength of different groups. Due to the epidemic control, physical therapists and older adult carry out OEP exercise plans at home. The outcome indicators show that family-based OEP can effectively prevent older adult from falling, improve the physical function and lower limb strength of family members, create a harmonious family atmosphere, and improve the subjective wellbeing of older adult (52). Walking movements in OEP projects, such as walking backward, zigzag walking, walking sideways, walking straight, climbing stairs, etc., continue to induce the activity of hip flexion muscle and isotonic flexion of the ankle, which may help to improve muscle strength and coordination (46). The research data for this part are shown in Table 3.

**Table 3:** Research data on OEP improving lower limb muscle strength.

**Researcher**	**Country**	**Sample size**	**Intervention time**	**Intervention mode**	**Test task**	**Research results**
Lee et al. (12)	Korea	*n* = 30; Age = 72.6 ± 2.67	12 weeks/3 times/60 min	①AR OEP; ② Yoga	Digital manual muscle tester	The knee flexion and ankle dorsiflexion strength in the OEP group increased, and the muscle strength of lower limbs in the OEP group was better than that in the yoga group
Liew et al. (45)	Malaysia	*n* = 67; Age ≥ 65	12 weeks/3 times/35 min	① Improved OEP; ② Routine nursing	Lower limb function test, hand dynamometer	The lower limb activity was improved and showed a cross effect, which improved its grip strength
Duckham et al. (47)	Britain	*n* = 142; Age ≥ 65	24 weeks/3 OEMs	① OEP family; ② Nursing + others	Bone densitometer; questionnaire investigation	There was no difference in bone mineral density between the two groups, and there was no increase in bone mineral density, but the muscle performance was improved, which was conducive to the prevention of fracture
Cheng et al. (50)	China	*n* = 20; Age ≥ 70	26 weeks/3 times/45 min	①OEP ② Daily activities	30-s standing test Lower limb muscle strength test	After OEP intervention, the muscle strength of the knee extensor, knee flexor, and ankle flexor was significantly improved
Jahanpeyma et al. (1)	turkey	*n* = 72, Age ≥ 65	12 weeks/3 times/45 min	①OEP ② None	Berg balance scale 30-s standing test	After the intervention, the number of sit and stand tests increased and the risk coefficient of falls decreased (*p* < 0.05)
Son et al. (8)	Korea	*n* = 46 Age = 65–79	12 weeks/twice/30 min	①OEP ② Tai Chi	Functional extension test One leg standing test	OEP group has a greater improvement in lower limb strength than the Tai Chi Group, which is conducive to the prevention of female elderly falls
Kocic (10)	Spain	*n* = 77 Age ≥ 65	26 weeks/3 times/35 min	①OEP ② Routine nursing	Functional independence measurement Timed standing walking test	Functional activity, lower limb muscle strength, and functional independence have significant effects, further delaying the progress of the disability
Mat et al. (23)	Singapore	*n* = 24; Age ≥ 60	unclear	①OEP ② Routine treatment	Stability test Lower limb strength test	Compared with the control group, it was found that the lower limb function of older adult after the intervention was enhanced and the effect of posture control was better
Park and Chang (51)	Korea	*n* = 8; Age ≥ 60	8 weeks/3 times/50 min	①OEP ② None	Energy efficiency meter Quality of life assessment scale	Due to the short sample size and intervention time, the impact on stroke patients is limited, but it is effective in preventing falls
Hager et al. (52)	Switzerland	*n* = 405; Age ≥ 65	unclear	①OEP ② None	Sitting test Four stages balance test	The risk of falls is reduced, to improve the quality of life of older adult
Leem et al. (46)	Korea	*n* = 30; Age ≥ 70	12 weeks/3 times/50 min	①OEP ② Action observation	Electronic muscle dynamometer	Compared with the control group, the activity of hip flexion muscle and isotonic flexion of the ankle in the OEP group increased, and the strength of lower limbs increased
Cederbom et al. (26)	Sweden	*n* = 119; Age ≥ 75	1 year, 30 min each time	①OEP ② None	Pain scale test Fall fear scale test	The test and follow-up study after the intervention showed that OEP exercise reduced lower limb pain and enhanced lower limb muscle strength

① Experimental group.

② Control group.

#### Effect of OEP on fall efficacy in older adult

Fall efficacy refers to the degree of self-efficacy that cannot be judged when participating in a certain activity. After older adult suffer from multiple falls, their fall efficiency and self-confidence decrease due to fear of falls, which increases the risk of falls and forms a vicious circle, further reducing the physical function of older adult. Physical exercise can improve social isolation, fear, and other related mental health problems (27).

The effect of exercise therapy should not be limited to the improvement of physiological outcome indicators. The psychological effect induced by exercise is also one of the important factors to reduce the risk of falls in older adult. Studies have shown that the inducing effect of OEP can enhance the balance and confidence of older adult and help eliminate the shadow and fear of falling. Domestic research using Internet + technology suggests that OEP can improve older adult's fear of falling, encourage older adult to exercise, and reduce the risk of falling (53). After 12 weeks of OEP intervention, psychological-related self-confidence problems will be effectively improved, and older adult's confidence in posture control will be enhanced (39). In addition, most of the fear of older adult comes from past fall history. Bjerk et al. (3) believe that in the positive benefits of OEP intervention, the psychological self-efficacy factor cannot be ignored, which plays an important role in reducing the risk of falls. OEP enhances the self-efficacy and happiness of older adult. The intervention of OEP indirectly improves the high-quality healthy life of older adult and meets the realistic requirements of older adult in their happy old age, rather than blindly extending the lifeline. This result was also verified in the interview survey. According to their many years of practical experience, 17 physiotherapists concluded that OEP can improve older adult's sense of fall efficacy, expand their social participation and promote older adult to better manage their daily life (54). An intervention study based on technical means believes that both online group exercise and social model OEP can improve the loneliness of older adult to a certain extent and improve the level of subjective wellbeing of older adult (28). However, some scholars believe that high cohesion group OEP is better than an individual exercise in reducing loneliness and improving subjective wellbeing, and online training for older adult living alone does not improve their loneliness (55). Before allowing older adult to participate in OEP, first carry out observation and learning for 20 min, control the actions of older adult, and then carry out OEP training, so that older adult can conduct self-assessment according to the observation and learning content, to increase their efforts and obtain a better sense of achievement (46).

The movement of OEP is not complex. Hale L and other (56) scholars took adults with slight intellectual impairment as the research object and found that it is not too difficult for patients to understand the content of OEP and can complete their training movements. Therefore, it is suggested that older adult with cognitive decline do not have too much psychological pressure and burden when completing. In addition to confirming the positive benefits of OEP for older adult, McMahon et al. (57) scholars also discussed the impact of interpersonal components in participating in training, including social environment, social support, cognitive self, self-encouraging behavior change, etc. these components will make older adult dare to exchange experience and share knowledge when participating in exercise, which will promote older adult's self-confidence in exercise and fall efficacy. Isolated elderly people in the context of the epidemic are prone to negative emotions such as depression and loneliness. OEP is used to reduce the negative effects caused by lack of exercise and significantly improve the depression and physical function of older adult (34). In addition, in the intervention study of elderly patients after knee arthroplasty, Liu Heng and other (25) scholars found that OEP significantly improved patients' fall efficiency and increased patients' self-confidence, and the exercise program can still exercise autonomously at home after patients are discharged from hospital to increase exercise benefits. However, there are data on OEP focusing on cognitive function, and it is unclear whether OEP plays several positive benefits in other cognitive fields. From the perspective of the impact of balance ability, after the exercise treatment of OEP, the dynamic and static balance ability of older adult has been significantly improved, which improves the body posture control ability of older adult, and then reduces the fall risk of older adult who are sedentary at home or engaged in daily outdoor activities. The research data for this part are shown in Table 4.

**Table 4:** Research data on the efficacy of OEP on falls in older adult.

**Researcher**	**Country**	**Sample size**	**Intervention time**	**Intervention mode**	**Test task**	**Research results**
Gu et al. (53)	China	*n* = 60, Age ≥ 65	24 weeks/3 times/30 min	①OEP ②Knowledge learning	Berg Balance Scale Energy efficiency meter test	The fall efficacy index was significantly improved, and OEP could improve the self-confidence of older adult
Chen et al. (38)	China	*n* = 60, Age ≥ 65	12 weeks/3 times/45 min	①OEP ② None	Specific activity balance confidence scale	The results showed that the self-confidence of older adult increased significantly, which was helpful to overcome the fear of falling
Baez et al. (28)	Russia	*n* = 40, Age ≥ 65	8 weeks, times/30–40 min	①OEP ② None	Multidimensional personality questionnaire Physical activity enjoyment scale	The results show that older adult like the sport, have high compliance, and improve their level of wellbeing
Nikitina et al. (55)	Russia	*n* = 44; Age ≥ 60	8 weeks/3 times/30 min	①OEP ② None	Physical activity enjoyment scale	After the intervention, the pre-test and post-test data found that the level of subjective wellbeing increased, but had little impact on loneliness
Leem et al. (46)	Korea	*n* = 30; Age ≥ 70	12 weeks/3 times/50 min	①OEP ② Action observation	Fear efficacy scale test	Compared with the control group, the walking speed, step frequency, step length and stride length of the OEP group increased significantly, indicating that the sense of self-efficacy was enhanced
McMahon et al. (57)	America	*n* = 308 Age ≥ 70	12 months, times/60 min	①OEP ② None	Social support questionnaire measurement Simple pain scale	Participating in exercise can increase the social participation of older adult, expand social support and improve self-efficacy
Chen et al. (32)	China	*n* = 62 Age ≥ 70	12 weeks/3 times/30 min	①OEP ② None	Geriatric depression scale Mental health survey	OEP improves the physical and psychological function of older adult with cognitive impairment
Heng et al. (25)	China	*n* = 32 Age ≥ 60	4 months, 7 times a week/30 min	①OEP ② Regular exercise	Single item problem method Energy efficiency meter	After the intervention, the fall efficacy and fear of elderly patients after knee arthroplasty decreased

① Experimental group.

② Control group.

## Discussion

To sum up, the positive effects of multi-component OEP on cognitive function, balance ability, lower limb muscle strength, and fall efficiency of older adult are consistent with the views of most literature, and it is considered that OEP is effective in preventing falls for older adult (10, 11, 14). From the perspective of the impact of cognitive function, it shows multifaceted positive effects on the cognitive level of older adult. OEP improves the processing speed, response inhibition, and other cognitive fields of older adult, and good executive function can predict and improve the exercise compliance of older adult, make them adhere to exercise and obtain good exercise benefits. However, most studies were single OEP exercise therapy and did not involve the combination of multiple balance training methods. Research shows that taking inspiratory muscle training as the auxiliary training method of OEP, the combination of the two therapies can better improve the balance function of older adult and the function of the inspiratory muscle of older adult (58). Supplementing OEP therapy with multi-sensory balance practice can also maximize the utility of balance ability (59). Therefore, OEP combined with other therapies may be more effective in balancing ability. In terms of the impact on lower limb muscle strength, OEP mainly reduces the risk of falls in older adult by improving muscle performance and enhancing muscle strength. The muscle strength of the lower limbs of older adult patients is significantly improved, especially the muscle strength of lower limbs is improved, which is affected by the aging of older adult patients. However, the physiological mechanism of OEP improving lower limb muscle strength is not clear, and whether it will increase bone mineral density and muscle cross-sectional area is unknown, but there is no doubt that it can delay muscle atrophy. From the perspective of the impact of older adult's fall efficiency, it is mainly to improve older adult's fall efficiency, increase their self-confidence, overcome the fear of previous falls, and enable them to complete some self-management things independently, so that they are full of confidence in their later life (54, 60), to achieve the purpose of reducing the risk of falls. The long-term sedentary elderly at home can alleviate the mood of depression and loneliness through OEP, expand social participation, enhance the feelings of their families and obtain the support of their families when exercising at home with their families, to improve the subjective wellbeing of older adult, which is very important for older adult who cannot go out of home due to physical factors.

To better participate in OEP exercise, practical problems in its operation also need to be further discussed, such as contraindications and exercise dose in special patients. Short-term training has little improvement on older adult with a history of falls, but only improves their physical performance. Therefore, older adult with a history of falls should actively participate in the exercise, reduce the risk of falls in time, and adhere to it for a long time to ensure the sustainability of the exercise effect. In the research with physiotherapists as interviewees, physiotherapists believe that OEP is an effective means to effectively prevent falls and improve physical function in older adult (61). The main reason is that OEP has strong applicability, simple project action, and diverse participation methods, older adult have little pressure on learning and training content and have good compliance, few adverse events, and high safety, which is very key. Therefore, the application of OEP in daily life exercise is also very feasible, not just limited to the field of clinical rehabilitation. Regardless of any sports event, the degree of exercise persistence is directly related to the exercise effect. After OEP training, telephone interviews, records, and other methods are adopted to encourage older adult to adhere to exercise, but the effect is very little, which is not enough to encourage them to adhere to exercise (62, 63). More means are needed to ensure the effective intensity and progress of the exercise. In the future, it can be combined with online special psychological counseling or cognitive intervention to achieve this purpose. The traditional way of one-to-one physical therapy for older adult seems to have been unable to meet the development of a rapidly aging society, and the economic burden is also an obstacle (11). Especially in rural areas with backward basic medical conditions, it is more difficult to implement the intervention plan. The combination of mobility organization and medical and health care, and the implementation of the OEP plan based on community conditions, have improved the physical function of older adult in rural areas and reduced the risk of falls (63–65). Previous literature has confirmed the effectiveness and reliability of OEP in the form of DVDs in rural areas, and older adult gain a sense of entertainment and happiness in social interaction. In addition, to allow special people to participate in the exercise, we need to improve the OEP content according to the actual situation. Therefore, we should design different training contents according to local conditions as far as possible to meet the needs of different patients, develop remote training and online guidance based on emerging technologies, and combine the traditional one-to-one OEP treatment for special elderly needs. Population aging is an inevitable trend of social development, and the problems caused by falls of older adult seem to be not only physical injuries but also have a great impact on the family and society. Therefore, how to effectively prevent falls of older adult is of great significance, especially in the post-epidemic era, after people's special experience of isolation and closure, they have a deeper understanding of the concept of a healthy life, In the future, we will pay more attention to the integration of sports into life. Based on the need for healthy aging, this paper summarizes the positive effects of OEP on preventing falls in older adult and effectively helping older adult live a healthy life in their later years. Therefore, this review has practical value and significance for the development of elderly health.

## Conclusion

Otago exercise has positive benefits in preventing falls in older adult, which can improve the cognitive function of older adult, enhance the muscle strength of lower limbs and the ability of dynamic and static balance, and then improve the gait stability and posture control ability of older adult; OEP is beneficial to improve the falling efficiency of older adult, help older adult overcome the fear of falling, and form a positive emotion of “exercise improve exercise,” to reduce the harm caused by sedentary behavior and the incidence of depression, and improve the subjective wellbeing of older adult. according to the review of this article, OEP exercise of 30–50 min three times a week is recommended as the exercise prescription for older adult to prevent falls. It is recommended that this exercise scheme be carried out as the daily regular activities of older adult at home or in the health care center. It is worth noting that special elderly people need to design exclusive exercise prescriptions to prevent the occurrence of adverse events.

## Limitations and further research directions

Most of the literature included in this paper is a single OEP exercise prescription, which cannot draw the positive benefit of OEP combined with other exercise methods to prevent falls in older adult; In addition, OPE lacks in-depth exploration of the deep mechanism of preventing falls in older adult. This is one of the limitations of this paper. The possible mechanisms of preventing falls in older adult are the improvement of muscle performance and cognitive function, but other mechanisms are not clear; To normalize this OEP movement in families, communities, and nursing homes in the post-epidemic era, and promote and improve it in combination with the actual situation of our country, it needs to be further discussed, and research in this area can be strengthened in the future.

## Data availability statement

The original contributions presented in the study are included in the article/supplementary material, further inquiries can be directed to the corresponding authors.

## Author contributions

YY collected and consulted literature and designed and wrote a review. KW, HL, JQ, YW, PC, TZ, and JL provide thesis writing guidance. JL is responsible for the evaluation and revision. All authors have read and agreed to the published version of the manuscript.

## Funding

This publication was funded by the National Social Science Foundation of China (Grant No: 19ZDA352).

## Conflict of interest

The authors declare that the research was conducted in the absence of any commercial or financial relationships that could be construed as a potential conflict of interest.

## Publisher's note

All claims expressed in this article are solely those of the authors and do not necessarily represent those of their affiliated organizations, or those of the publisher, the editors and the reviewers. Any product that may be evaluated in this article, or claim that may be made by its manufacturer, is not guaranteed or endorsed by the publisher.

## References

[1] Jahanpeyma P Kayhan Koçak FÖ Yildirim Y Sahin S Senuzun Aykar F. Effects of the Otago exercise program on falls, balance, and physical performance in older nursing home residents with high fall risk: a randomized controlled trial. Eur Geriatr Med. (2021) 12:107–15. 10.1007/s41999-020-00403-1 33237565

[2] Liston M Genna G Maurer C Kikidis D Gatsios D Fotiadis D . Investigating the feasibility and acceptability of the HOLOBalance system compared with standard care in older adults at risk for falls: study protocol for an assessor blinded pilot randomised controlled study. BMJ Open. (2021) 11:e039254. 10.1136/bmjopen-2020-039254 33579762 PMC7883859

[3] Bjerk M Brovold T Skelton DA Bergland A. A falls prevention programme to improve quality of life, physical function and falls efficacy in older people receiving home help services: study protocol for a randomised controlled trial. BMC Health Serv Res. (2017) 17:559. 10.1186/s12913-017-2516-5 28806904 PMC5556992

[4] Qi Y Chang H Liu D Bao S Weicu. Occurrence and influencing factors of falls among older adult in Dalian community. Chin J Gerontol. (2021) 41:2866–9.

[5] Yang G Yang W. Influencing factors of compliance with fall prevention measures in elderly patients. Chin J Gerontol. (2020) 40:4679–81.

[6] QianXX ChauPH KwanCW LouV LeungA HoM . Investigating risk factors for falls among community-dwelling older adults according to WHO's risk factor model for falls. J Nutr Health Aging. (2021) 25:425–32. 10.1007/s12603-020-1539-533786558

[7] Albornos-Muñoz L Moreno-Casbas MT Sánchez-Pablo C Bays-Moneo A Fernández-Domínguez JC Rich-Ruiz M . Efficacy of the Otago Exercise Programme to reduce falls in community-dwelling adults aged 65-80 years old when delivered as group or individual training. J Adv Nurs. (2018) 74:1700–11. 10.1111/jan.13583 29633328

[8] Son NK Ryu YU Jeong HW Jang YH Kim HD. Comparison of 2 different exercise approaches: Tai Chi Versus Otago, in community-dwelling older women. J Geriatr Phys Ther. (2016) 39:51–7. 10.1519/JPT.0000000000000042 25760277

[9] Wu P Ke Y Ho C-Y Yu W. Exercise prescription for preventing falls in older adult. Beishi Med J. (2020) 17:20–30. 10.6200/TCMJ.202003_17(1).0003

[10] Kocic M Stojanovic Z Nikolic D Lazovic M Grbic R Dimitrijevic L . The effectiveness of group Otago exercise program on physical function in nursing home residents older than 65 years: a randomized controlled trial. Arch Gerontol Geriatr. (2018) 75:112–8. 10.1016/j.archger.2017.12.001 29241091

[11] Shubert TE Smith ML Jiang L Ory MG. Disseminating the Otago Exercise Program in the United States: perceived and actual physical performance improvements from participants. J Appl Gerontol. (2018) 37:79–98. 10.1177/0733464816675422 27794055

[12] Lee J Yoo HN Lee BH. Effects of augmented reality-based Otago exercise on balance, gait, and physical factors in elderly women to prevent falls: a randomized controlled trial. J Phys Ther Sci. (2017) 29:1586–9. 10.1589/jpts.29.1586 28931993 PMC5599826

[13] Aranda-Reneo I Albornos-Muñoz L Rich-Ruiz M Cidoncha-Moreno MÁ Pastor-López Á Moreno-Casbas T . Cost-effectiveness of an exercise programme that provided group or individual training to reduce the fall risk in healthy community-dwelling people aged 65-80: a secondary data analysis. Healthcare. (2021) 9:714. 10.3390/healthcare9060714 34200873 PMC8230501

[14] Chiu HL Yeh TT Lo YT Liang PJ Lee SC. The effects of the Otago Exercise Programme on actual and perceived balance in older adults: a meta-analysis. PLoS ONE. (2021) 16:e0255780. 10.1371/journal.pone.0255780 34358276 PMC8345836

[15] Martins AC Santos C Silva C Baltazar D Moreira J Tavares N. Does modified Otago Exercise Program improves balance in older people? A systematic review. Prev Med Rep. (2018) 11:231–9. 10.1016/j.pmedr.2018.06.015 30210995 PMC6129967

[16] Campbell AJ Robertson MC Gardner MM Norton RN Buchner DM. Falls prevention over 2 years: a randomized controlled trial in women 80 years and older. Age Ageing. (1999) 28:513–8. 10.1093/ageing/28.6.513 10604501

[17] Campbell AJ Robertson MC. Comprehensive approach to fall prevention on a national level: New Zealand. Clin Geriatr Med. (2010) 26:719–31. 10.1016/j.cger.2010.06.004 20934618

[18] Araújo F Nogueira MN Silva J Rego S. A technological-based platform for risk assessment, detection, and prevention of falls among home-dwelling older adults: protocol for a Quasi-experimental study. JMIR Res Protoc. (2021) 10:e25781. 10.2196/25781 34387557 PMC8391727

[19] Gu B Zhang Q Ma Q Yu H Zhang L Yu Q. Research progress on the application of Otago exercise at home and abroad. Nurs Res. (2019) 33:3555–8.

[20] Shubert TE Smith ML Ory MG Clarke CB Bomberger SA Roberts E . Translation of the Otago exercise program for adoption and implementation in the United States. Front Public Health. (2015) 2:152. 10.3389/fpubh.2014.00152 25964899 PMC4410425

[21] Shubert TE Chokshi A Mendes VM Grier S Buchanan H Basnett J . Stand tall-A virtual translation of the Otago exercise program. J Geriatr Phys Ther. (2020) 43:120–7. 10.1519/JPT.0000000000000203 29958232

[22] Pei Z Wang M Meng X. Meta-analysis of the intervention effect of Otago exercise program on falls in stroke patients. Rehabil J. (2019) 29:60–6.

[23] Mat S Ng CT Tan PJ Ramli N Fadzli F Rozalli FI . Effect of modified Otago exercises on postural balance, fear of falling, and fall risk in older fallers with knee osteoarthritis and impaired gait and balance: a secondary analysis. PM R. (2018) 10:254–62. 10.1016/j.pmrj.2017.08.405 28827207

[24] Liu-Ambrose T Davis JC Falck RS Best JR Dao E Vesely K . Exercise, processing speed, and subsequent falls: a secondary analysis of a 12-month randomized controlled trial. J Gerontol A Biol Sci Med Sci. (2021) 76:675–82. 10.1093/gerona/glaa239 33225343

[25] Liu H Ji D Chi X Gu X Bai C Zhao Q. Effects of OEP on balance ability and fear of falling in elderly patients after knee replacement. Chin Nurs Manag. (2019) 19:133–8.

[26] Cederbom S Arkkukangas M. Impact of the fall prevention Otago Exercise Programme on pain among community-dwelling older adults: a short- and long-term follow-up study. Clin Interv Aging. (2019) 14:721–6. 10.2147/CIA.S200188 31118594 PMC6498390

[27] VanRavenstein K Davis BH. When more than exercise is needed to increase chances of aging in place: qualitative analysis of a telehealth physical activity program to improve mobility in low-income older adults. JMIR aging. (2018) 1:e11955. 10.2196/11955 31518250 PMC6715103

[28] Baez M Khaghani Far I Ibarra F Ferron M Didino D Casati F. Effects of online group exercises for older adults on physical, psychological and social wellbeing: a randomized pilot trial. PeerJ. (2017) 5:e3150. 10.7717/peerj.3150 28392983 PMC5384569

[29] Blackwood J Shubert T Forgarty K Chase C. Relationships between performance on assessments of executive function and fall risk screening measures in community-dwelling older. Adults J Geriatr Phys Ther. (2016) 39:89–96. 10.1519/JPT.0000000000000056 26050194

[30] Jehu DA Davis JC Madden K Parmar N Liu-Ambrose T. Minimal clinically important difference of executive function performance in older adults who fall: a secondary analysis of a randomized controlled trial. Gerontology. (2022) 68:771–9. 10.1159/000518939 34657043

[31] Huang J Ou Y. Nursing effects of Otago exercise training on neurological and motor function of stroke patients. J Taishan Med Coll. (2020) 41:787–8.

[32] Chen X Zhao L Liu Y Zhou Z Zhang H Wei D . Otago exercise programme for physical function and mental health among older adults with cognitive frailty during COVID-19: a randomised controlled trial. J Clin Nurs. (2021). 10.1111/jocn.15964. [Epub ahead of print]. 34289524 PMC8447213

[33] Davis JC Khan K Mansournia MA Khosravi A Rhodes RE Chan P . A 'case-mix' approach to understand adherence trajectories for a falls prevention exercise intervention: a longitudinal cohort study. Maturitas. (2021) 147:1–6. 10.1016/j.maturitas.2021.02.004 33832641

[34] Davis JC Rhodes RE Khan KM Mansournia MA Khosravi A Chan P . Cognitive function and functional mobility predict exercise adherence in older adults who fall. Gerontology. (2021) 67:350–6. 10.1159/000513452 33631742

[35] Liu-Ambrose T Donaldson MG Ahamed Y Graf P Cook WL Close J . Otago home-based strength and balance retraining improves executive functioning in older fallers: a randomized controlled trial. J Am Geriatr Soc. (2008) 56:1821–30. 10.1111/j.1532-5415.2008.01931.x 18795987

[36] Liu J Zhang Q Yu L. Application of Otago exercise intervention combined with music therapy in the rehabilitation period of stroke patients. Qilu Nurs Jo. (2021) 27:90–2.

[37] Shao C. Study on the Effect of OEP on Cognitive Function of Stroke Patients [D]. Shandong: Shandong University (2019).

[38] Chen X Xiao Y Pei X. The influence of OEP on the balance ability and balance confidence of older adult in nursing institutions. China Rehabil Theory Pract. (2019) 25:1193–6.

[39] Beato M Dawson N Svien L Wharton T. Examining the effects of an Otago-Based Home Exercise Program on falls and fall risks in an assisted living facility. J Geriatr Phys Ther. (2019) 42:224–9. 10.1519/JPT.0000000000000190 29698252

[40] Knott S Hollis A Jimenez D Dawson N Mabbagu E Beato M. Efficacy of traditional physical therapy versus Otago-Based Exercise in fall prevention for ALF-residing older adults. J Geriatr Phys Ther. (2021) 44:210–8. 10.1519/JPT.0000000000000285 33534336

[41] Phu S Vogrin S Al Saedi A Duque G. Balance training using virtual reality improves balance and physical performance in older adults at high risk of falls. Clin Interv Aging. (2019) 14:1567–77. 10.2147/CIA.S220890 31695345 PMC6717859

[42] Wang L Zhang T Zhang Q. The effect of Otago exercise on the debilitating elderly in nursing homes. J Nurs. (2019) 34:12–5.

[43] Feng H Zou Z Zhang Q Wang L Ouyang YQ Chen Z . The effect of the group-based Otago exercise program on frailty among nursing home older adults with cognitive impairment. Geriatr Nurs. (2021) 42:479–83. 10.1016/j.gerinurse.2021.02.012 33714906

[44] An Q Jia S Zhang Y Wang S Hu B Feng C. Construction and implementation of an evidence-based group fall prevention OEP scheme for older adult. J Nurs. (2019) 34:83–6.

[45] Liew LK Tan MP Tan PJ Mat S Majid LA Hill KD . The Modified Otago Exercises prevent grip strength deterioration among older fallers in the Malaysian falls assessment and intervention trial (MyFAIT). J Geriatr Phys Ther. (2019) 42:123–9. 10.1519/JPT.0000000000000155 29381526

[46] Leem SH Kim JH Lee BH. Effects of Otago exercise combined with action observation training on balance and gait in the old people. J Exerc Rehabil. (2019) 15:848–54. 10.12965/jer.1938720.360 31938708 PMC6944869

[47] Duckham RL Masud T Taylor R Kendrick D Carpenter H Iliffe S . Randomised controlled trial of the effectiveness of community group and home-based falls prevention exercise programmes on bone health in older people: the ProAct65+ bone study. Age Ageing. (2015) 44:573–9. 10.1093/ageing/afv055 25906791 PMC4476850

[48] Ruan Q D'onofrio G Wu T Greco A Sancarlo D Yu Z. Sexual dimorphism of frailty and cognitive impairment: potential underlying mechanisms (Review). Mol Med Rep. (2017) 16:3023–33. 10.3892/mmr.2017.6988 28713963

[49] Paolucci EM Loukov D Bowdish D Heisz JJ. Exercise reduces depression and inflammation but intensity matters. Biol Psychol. (2018) 133:79–84. 10.1016/j.biopsycho.2018.01.015 29408464

[50] Cheng YC Liao YC Hsieh LY. Effects of the Otago Exercise Program on lower extremity strength in residents of a long-term care institution. J Nurs. (2020) 67:48−55. 32495329 10.6224/JN.202006_67(3).07

[51] Park Y Chang M. Effects of the Otago exercise program on fall efficacy, activities of daily living and quality of life in elderly stroke patients. J Phys Ther Sci. (2016) 28:190–3. 10.1589/jpts.28.190 26957755 PMC4756001

[52] Mittaz Hager AG Mathieu N Lenoble-Hoskovec C Swanenburg J de Bie R Hilfiker R. Effects of three home-based exercise programmes regarding falls, quality of life and exercise-adherence in older adults at risk of falling: protocol for a randomized controlled trial. BMC Geriatr. (2019) 19:13. 10.1186/s12877-018-1021-y 30642252 PMC6332592

[53] Gu Y Shen Y Yu X Zhu Y. Application effect of OEP in elderly people who are afraid of falling. Nurs Res. (2020) 34:1253−6.

[54] Cederbom S Bjerk M Bergland A. A qualitative study exploring physical therapists' views on the Otago Exercise Programme for fall prevention: a stepping stone to “age in place” and to give faith in the future. Physiother Theory Pract. (2022) 38:132–40. 10.1080/09593985.2020.1731895 32090667

[55] Nikitina S Didino D Baez M Casati F. Feasibility of virtual tablet-based group exercise among older adults in siberia: findings from two pilot trials. JMIR mHealth uHealth. (2018) 6:e40. 10.2196/mhealth.7531 29487045 PMC5849795

[56] Hale L Vollenhoven E Caiman L. Feasibility and acceptability of Otago Exercise Programme and prevention of falls for adults with intellectual disability: a multiple case study design. Int J Ther Rehabil. (2019) 26:1–15. 10.12968/ijtr.2018.0054

[57] McMahon SK Lewis BA Guan W Wyman JF Rothman AJ. Community-based intervention effects on older adults' physical activity and falls: Protocol and rationale for a randomized optimization trial (Ready Steady3.0). Contemp Clin Trials. (2021) 101:106238. 10.1016/j.cct.2020.106238 33285280 PMC8266260

[58] Ferraro FV Gavin JP Wainwright TW McConnell AK. Comparison of balance changes after inspiratory muscle or Otago exercise training. PLoS ONE. (2020) 15:e0227379. 10.1371/journal.pone.0227379 31978126 PMC6980667

[59] Liston MB Alushi L Bamiou DE Martin FC Hopper A Pavlou M. Feasibility and effect of supplementing a modified OTAGO intervention with multisensory balance exercises in older people who fall: a pilot randomized controlled trial. Clin Rehabil. (2014) 28:784–93. 10.1177/0269215514521042 24776526

[60] Tang L Yue L Liu C. The effect of Otago exercise on the fear of falling and balance ability in discharged Parkinson's patients. Chin J Rehabil Med. (2016) 31:1383–5.

[61] Kemmler W von Stengel S Engelke K Häberle L Kalender WA. Exercise effects on bone mineral density, falls, coronary risk factors, and health care costs in older women: the randomized controlled senior fitness and prevention (SEFIP) study. Arch Intern Med. (2010) 170:179–85. 10.1001/archinternmed.2009.499 20101013

[62] Clemson L Singh MF Bundy A Cumming RG Weissel E Munro J . LiFE Pilot Study: a randomised trial of balance and strength training embedded in daily life activity to reduce falls in older adults. Aust Occup Ther J. (2010) 57:42–50. 10.1111/j.1440-1630.2009.00848.x 20854564

[63] Yamada M Higuchi T Nishiguchi S Yoshimura K Kajiwara Y Aoyama T. Multitarget stepping program in combination with a standardized multicomponent exercise program can prevent falls in community-dwelling older adults: a randomized, controlled trial. J Am Geriatr Soc. (2013) 61:1669–75. 10.1111/jgs.12453 24001116

[64] Guan X Zhu X Liu J. Effects of yoga exercise on balance ability and fear of falling in early Parkinson's patients. Nurs Res. (2017) 31:1274–6.

[65] Tuvemo Johnson S Anens E Johansson AC Hellström K. The Otago Exercise Program with or without motivational interviewing for community-dwelling older adults: a 12-month follow-up of a randomized, controlled trial. J Appl Gerontol. (2021) 40:289–99. 10.1177/0733464820902652 32114877 PMC7874375

